# Human disturbance alters the foraging and spatiotemporal activity of a large carnivore

**DOI:** 10.1007/s00442-025-05752-x

**Published:** 2025-06-26

**Authors:** Gonzalo Barceló, Emiliano Donadio, Mathew W. Alldredge, Jonathan N. Pauli

**Affiliations:** 1 Department of Forest and Wildlife Ecology, University of Wisconsin-Madison, 1630, Linden Dr., Madison, WI 53706 USA; 2 Departamento de Ciencias, Facultad de Artes Liberales, Universidad Adolfo Ibáñez, Santiago, Chile; 3 Fundación Rewilding Argentina, Scalabrini Ortiz 3355 4J, Buenos Aires, CP 1425 Argentina; 4 Colorado Parks and Wildlife, Fort Collins, CO 80526 USA

**Keywords:** Carnivore movement, Global change, Human impact, Niche plasticity, *Puma concolor*, Trophic specialization

## Abstract

Carnivore recovery is often promoted to restore ecosystem functioning. However, human-disturbed landscapes can alter the behavior and role of returning carnivores. To evaluate how different dimensions of a carnivore niche are affected across a gradient of human disturbance, we studied 83 pumas (*Puma concolor*) at seven populations in North and South America. We hypothesized that pumas inhabiting sites with high human disturbance would modify their niche by reducing space use, becoming more nocturnal and diversifying their diets. We quantified how landscape features affected puma home ranges, movement paths, diel activity, and step selection. Using stable isotopes, we quantified individual diet and dietary specialization, and population dietary niche width. Pumas decreased their movement rate with increasing human disturbance while some evidence indicates home ranges were reduced. Unexpectedly, diel activity was unaffected by human disturbance, but pumas decreased movement more during the day in areas with high disturbance. Similarly, pumas avoided highly disturbed areas during the day, but that avoidance was low at night. Finally, individual dietary specialization decreased because of pumas reduced consumption of native ungulates with increasing disturbance, although without changes in the population niche width. Responses to human disturbance were generally consistent across sites, with pumas adjusting their temporal, spatial, and foraging axes to decrease encounters with humans. Our results suggest that human-disturbed landscapes across regions alter the primary niche axes of pumas to construct a new realized niche in human landscapes, which may have important consequences for their ecological interactions and the functional role of this large carnivore.

**Supplementary Information:**

The online version contains supplementary material available at 10.1007/s00442-025-05752-x.

## Introduction

Accelerated environmental changes in the Anthropocene are restructuring both the stage and actors involved in the ecological play, even to the point of creating novel ecosystems (Radeloff et al. 2015). Human conversion of natural landscapes to agricultural and urban areas has resulted in habitat loss and imposed physical barriers to animal movement (Crooks et al. 2011; Tucker et al. 2018). Human actions also alter the behavior of animals (Frey et al. 2020), which have been documented as becoming increasingly nocturnal (Gaynor et al. 2018; Suraci et al. 2019). Additionally, human-disturbed landscapes often feature environments with altered species composition (Luck and Daily 2003) and allochthonous subsidies (Manlick and Pauli 2020), which can transform biotic interactions (Gilbert et al. 2022). The temporal, spatial, and trophic aspects of an organism comprise three major axes of its ecological niche; the modification of any one of these axes can result in altered functional roles (Ritchie et al. 2012; Kuijper et al. 2016; Pauli et al. 2018). While it is increasingly recognized that animals possess sufficient plasticity to persist within human-disturbed landscapes, the different niche axes have rarely been assessed simultaneously. Thus, how shifting niche components may lead to a new realized niche in novel conditions is largely unknown.

Carnivores are currently recolonizing part of their historical distribution due to conservation efforts, cultural acceptance, and legal protection; however, they are returning to landscapes modified and inhabited by humans (Athreya et al. 2013; Chapron et al. 2014; Pauli et al. 2018). Many ecologists and conservationists advocate for carnivore recovery with the hope of maintaining or restoring their strong top-down regulations as part of ecosystem restoration (Miller et al. 2001; Fraser et al. 2015; Villar 2023). However, carnivores within these novel landscapes may feature different functional roles and have unpredictable effects on biological communities (Ritchie et al. 2012; Kuijper et al. 2016; Pauli et al. 2018). Indeed, carnivores in novel landscapes present altered behavior in the use of space and time (Tucker et al. 2018; Gaynor et al. 2018), a more generalist diet (Manlick and Pauli 2020) and can have a reduced or increased top-down regulation over prey (Wallach et al. 2010; Smith et al. 2017).

Carnivores tend to avoid human-disturbed areas or pass through them, selecting small areas of natural habitats within these landscapes (Bateman and Fleming 2012; Šálek et al. 2015; Tucker et al. 2018; Whittington et al. 2022). Concurrently, human landscapes can provide food subsidies, such as food waste, bait, supplemental feed, and livestock, increasing the abundance of synanthropic species (Shochat et al. 2010; West et al. 2016; Kirby et al. 2017; Manlick et al. 2020; Penteriani et al. 2021). These subsidies can reduce the time spent searching for prey and the distances they travel while hunting (Parsons et al. 2022). To avoid human encounters, many carnivores increase their nocturnal activity, which depends more on the intensity of human development rather than just the presence of humans (Gaynor et al. 2018; Barocas et al. 2018). This nocturnality enables carnivores to navigate disturbed landscapes while minimizing lethal encounters (Mills et al. 2023). However, a shift in the time of activity alongside the altered assemblage of species in human-disturbed landscapes can lead to modifications in biotic interactions, potentially reshaping predator–prey and intraguild dynamics (Curras et al. 2022; Gilbert et al. 2022).

Carnivores can, however, exhibit varying and sometimes contrasting responses to human-disturbed landscapes, influenced by the type of disturbance and the species involved (Lowry et al. 2013; Sévêque et al. 2020). In agricultural areas, some carnivores, like tigers (*Panthera tigris*), increase their movement rates and home ranges to find suitable cover despite human presence (Habib et al. 2021; Naha et al. 2021). Similarly, linear features such as roads with low traffic volumes or transmission lines are used by carnivores, increasing their movement rate (Dickie et al. 2017). This increased movement is often associated with carnivores abandoning carcasses prematurely when human activity disrupts foraging on kills (Smith et al. 2016), which erodes the benefit of hunting large prey and may induce a shift to smaller prey or increase their kill rate (Smith et al. 2015, 2016). Additionally, a shift to nocturnality is not universal, as some carnivores maintain their usual activity patterns by being selective about the spaces they use when humans are present (Van Cleave et al. 2018). In urban areas, high human density and traffic lead to greater avoidance behavior (Kautz et al. 2021). Rural areas typically feature lower human activity, which leads to less spatial avoidance by carnivores, although conflicts due to livestock presence persist (Ohrens et al. 2016; Naha et al. 2021). Previous studies, though, have not disentangled the potentially differing roles of human disturbance types or investigated such responses simultaneously in different populations of the same species. Furthermore, there has been a general bias towards research in North America in relation to other parts of the globe, particularly more than tripling the research in human-carnivores relations done in South America (Lozano et al. 2019).

**Fig. 1:**
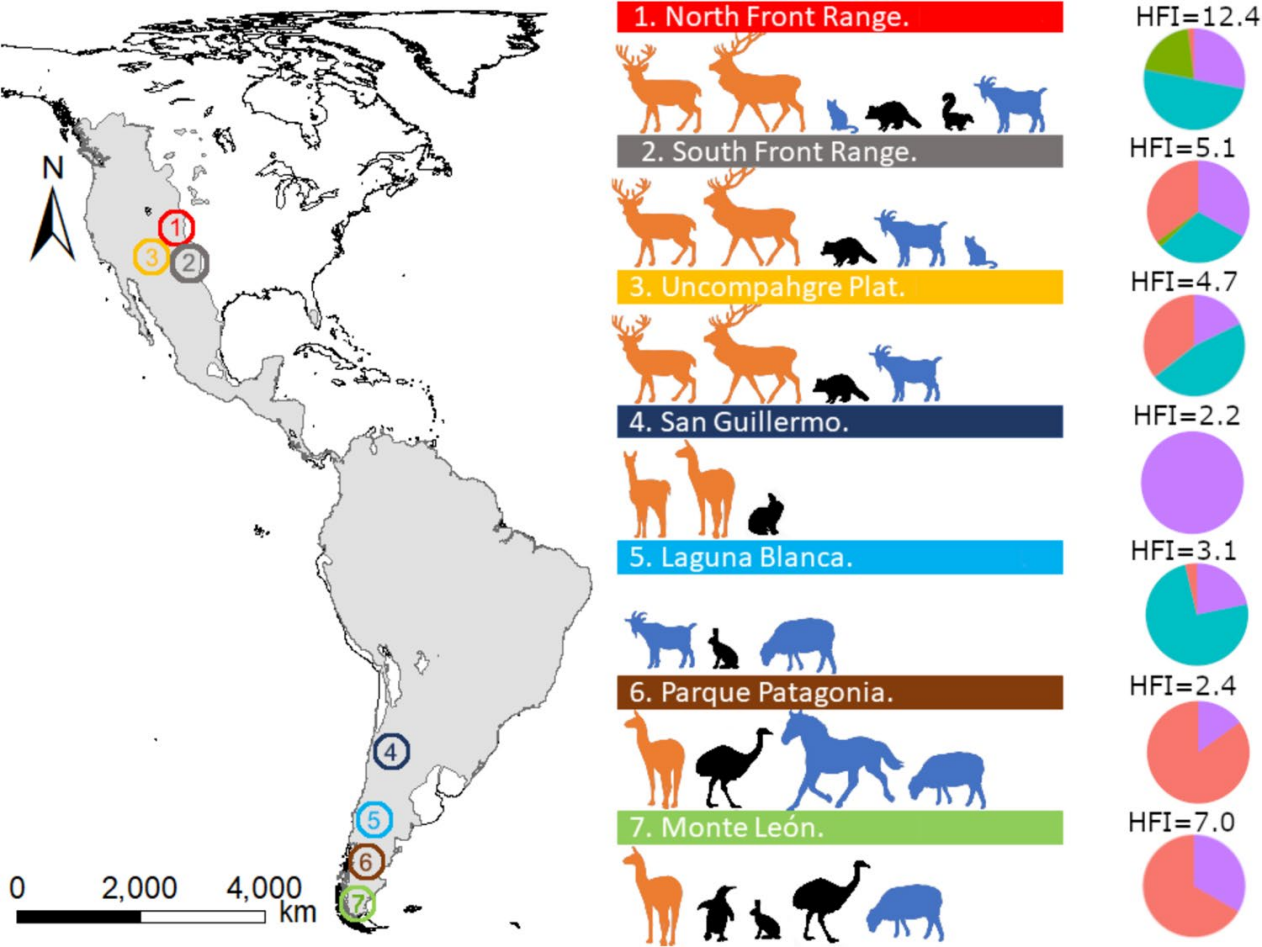
Puma (*Puma concolor*) current distribution in the Americas (light grey, IUCN 2022) showing the seven study sites with the corresponding prey species found in kill-site investigations and the average human footprint index (HFI) for the overlapping home range of the population in each site. Prey indicate availability accordingly to kill-site investigations and is categorized as domestic, including livestock and pets (blue; goat, cat, sheep, horse); large wild animals, corresponding to native ungulates (orange; mule deer, elk, vicuña, guanaco); and small wild prey, which include wild prey weighing less than 20 kg (black; raccoon, skunk, viscacha, European hare, rhea, Magellanic penguin). The pie chart reflects the composition of the HFI of each study site, categorized as agricultural (light red), road proximity (purple), human density (cyan), and built infrastructure (green)

Pumas (*Puma concolor*) range from the Yukon in Canada to the southernmost continental South America (Fig. 1). Pumas are generalist predators and exhibit great behavioral plasticity; thus, they occur in human-disturbed landscapes when hunting pressure is reduced, exurban areas (Moss et al. 2016a) and agroecosystems (Azevedo et al. 2018). Currently, pumas are returning to many parts of their historic range (Mazzolli 2012; O′Neil et al. 2014); however, it is still unclear what human landscape variables most strongly interact with puma niche use. It appears, though, that rural areas are perceived by pumas as less hostile compared to exurban and urban areas, which are frequently avoided (Burdett et al. 2010). Pumas inhabiting temperate latitudes generally feed upon large ungulates (Iriarte et al. 1990; Karandikar et al. 2022) but in human landscapes they incorporate non-native prey in their diets (Novaro et al. 2000; Moss et al. 2016a). In North America, pumas in urban-wildland interfaces consume synanthropic prey (Moss et al. 2016b; Wang et al. 2017); while in South America, pumas frequently use agricultural lands, where they can prey on livestock, exacerbating human-wildlife conflicts (Guerisoli et al. 2021). However, an analysis quantifying puma niche as a function of human disturbance across their distribution range utilizing common measures has not yet been conducted (but see Karandikar et al. 2022 and LaBarge et al. 2022 for reviews). Consequently, elucidating the trophic, spatial and temporal responses of pumas in varying ecological context can help to identify their functional role and the scenarios in which recovery may bring the ecosystem services sought or potentially increase conflict with human interests.

Given the increasing occurrence of pumas in human-disturbed landscapes (Moss et al. 2016a; Pereira et al. 2020) due to recolonization of former ranges (Mazzolli 2012; Gigliotti et al. 2019) and human expansion (Zalles et al. 2021), there is uncertainty around the consequences for pumas on these novel landscapes and the transformation of their spatial–temporal and trophic niche. To test how pumas respond to human disturbance, we quantified the individual diets of marked pumas, their movement patterns and their selection of habitat in different populations inhabiting temperate zones of North and South America with varying degrees of human disturbance. We hypothesized that pumas inhabiting human-disturbed landscapes would present differential trophic, spatial and temporal behaviors when compared to pumas in mostly pristine areas. Specifically, we predicted that in human-disturbed landscapes, the niche of pumas will be modified in at least one dimension exhibiting broader dietary niches with reduced specialization, reduced movement and smaller home ranges, and increased nocturnality.

## Methods

### Study sites

Pumas were sampled in seven sites distributed across the temperate latitudes of the Americas (Fig. 1): (1) Northern Front Range, CO (40° 0′ N, 106° 2′ W) is an urban-wildland interface featuring high human density and development. (2) Southern Front Range, CO (38° 26 N, 105° 13′ W); corresponds to a less populated urban-wildland interface. (3) Uncompahgre Plateau, CO (38º 20′ N, 108º 05′ W) corresponds primarily to undeveloped land, with few developments only around the perimeter of the study site. (4) San Guillermo National Park, Argentina (29° 13′ S, 69°31′ W) located in the Andean high plateau, exhibits virtually no human presence and a near-pristine landscape. (5) Laguna Blanca National Park, Argentina (39° 02′ S, 70° 21′ W) in the Patagonian steppe is protected but undergoes heavy grazing by livestock, especially sheep and goats; shepherds live in isolated houses scattered around the park. (6) Patagonia Park, Argentina (46° 36′ S, 71° 24′ W) also in the Patagonian steppe has little human development and past land use was primarily for rangelands until 2019. (7) Monte León National Park, Argentina (50° 06′ S, 68° 54′ W) is located on the Atlantic coast of the Patagonian steppe, formerly used as rangeland but transformed into a national park in 2006.

### Study animals

Pumas were captured as part of associated studies using snares, hound tracking (Moss et al. 2016a; Barceló et al. 2022) or cage traps (Smith et al. 2019) and anesthetized to collect hair samples (*n* = 83) and fit GPS collars (*n* = 57) following the protocols of Colorado Parks and Wildlife (ACUC # 09–2018, 16–2008 and 08–2004), Argentine National Park Administration (permits DRC265, DRPA162 and DRPN1678), and Consejo Agrario Provincial, Santa Cruz (dispositions 19/2018, 09/2019, 37/2019, and 4/2020). Pumas were captured over a period of three years within each site (See Supplementary Data “Capture” for details). Hair and feathers from prey were collected during each capture season at each site from carrion and road kills found opportunistically and while walking transects. Samples were collected with forceps and deposited in coin paper envelopes in a dry environment until further processing in the lab. Domestic prey samples were collected from the Northern Front Range to represent domestic species for all sites in North America. Prey included in the analysis for each study site was based on kill-site investigations, accounting for at least 10% of the population kills or 20% of individual kills (see Supplementary Data, “Prey” for details). There were two sites where GPS-collars were not deployed (Laguna Blanca and Uncompahgre Plateau). GPS collars collected locations at a fix rate of 8 per day in most sites but 6 per day in the Southern Front Range, over at least six months until one year (see Supplementary Data “Capture” for details). GPS points fixed with less than three satellites or a dilution of precision higher than six were eliminated (Frair et al. 2010).

### Home ranges

To estimate the home ranges of the GPS collared individual, we used the minimum convex polygon method with 90% of the data using the package *adehabitat* (Calenge 2006) within the program R (version 4.4.1; R Core Team 2024). The home range obtained from each individual was used to extract the mean value of the Human Footprint Index (HFI; Venter et al. 2016), where higher values represent a more disturbed landscape. We used HFI to represent human disturbance because it is an index at a global scale that allows comparisons between Argentina and the USA. The HFI also represents a composite of human disturbance elements (human density, roads, cropland, pasturelands, railways, night-time lights and built environments) that can be decomposed to address each element independently. We evaluated the response of the home ranges area to HFI using a linear, mixed-effects model accounting for sex and age class (adult-subadult) as fixed effects and site as a random effect using *glmmTMB* (Brooks et al. 2017) package in R and log transforming the home range to avoid the right-skewed distribution.

### Movement

We evaluated the movement rate and the turning angles of each step as a measure of the activity of pumas where a higher movement rate indicates higher activity and angles closer to zero indicate more direct traveling. Because pumas at different sites were outfitted with either 3- or 4 h GPS fix intervals, we standardized the movement by time and analyzed movement rate (m/hr) (Johnson et al. 2006; Leclerc et al. 2021). Then, we transformed the movement rate using a natural logarithm. We evaluated these variables in response to HFI, the time of the day, and their interaction using a linear mixed-effect model accounting site and ID as random effects using R software with package *lme4* (Bates et al. 2015) for the movement rate and using *brms* package (Bürkner 2017) with a von Mises regression model with a tan-half link for turning angles. We defined the time of day as *day* (1 h after sunrise and one before sunset) or *night* (1 h after sunset and 1 h before sunrise).

### Temporal activity

To quantify diel activity patterns for pumas, we calculated the mean movement rate of individuals between successive points as the mean distance traveled per hour. We examined diel movement rates for the five sites with collared individuals by fitting generalized additive mixed models (GAMM) with a cyclic spline and a random intercept for individuals using the *mgcv* package in R (Wood 2001; Kohl et al. 2018). Finally, we calculated the nocturnality value for each individual as the distance traveled during the night divided by the total distance traveled (i.e., the proportion of distance traveled during the night). Then, we fit a mixed-effects beta regression model with a logit link using nocturnality as the response variable, the HFI of the home range as the predictor, and a random intercept for the site using package *glmmTMB* (Brooks et al. 2017) in R software.

### Space use and selection

To account for the environmental variables and the human influence over the puma movement pattern, we used an integrative Step-Selection Analysis (iSSA, Avgar et al. 2016). iSSA can estimate resource selection parameters concurrently with the movement pattern of the animal, allowing joint inference on both processes. As iSSA requires consistent fix intervals, we fractioned the whole individual puma trajectory into several sets with constant intervals between fixed locations, with at least 3 sequential locations. Although selection is scale dependent, the 3 h and 4 h schedules represent a similar timescale that allows comparisons between individual selection coefficients (Northrup et al. 2016; Tsalyuk et al. 2019). For each step between two successive locations, we generated ten random steps with the *amt* package in R (Signer et al. 2019), using a gamma distribution for the step lengths and von Mises distribution for the turning angles (Lund et al. 2017). We also categorized each step as day or night based on the sunset and sunrise for each given location and time of the year. As environmental covariates for the iSSA, we used the components of the HFI to address the different types of human disturbance (Venter et al. 2016), specifically: road proximity, human density, agricultural intensity, the extent of built human infrastructure and the total HFI. We also considered natural variables that have previously been shown to impact the movement and behavior of pumas. Specifically, we included elevation, roughness and slope (Danielson and Gesch 2011) as they represent accessibility of the terrain (Dunford et al. 2020) as well as hunting cover for predators (Smith et al. 2019); distance to water (Kummu et al. 2011), as an attractant for both prey and predators (Crosmary et al. 2012); and cumulative, minimum and variability of the gross primary productivity (GPP; Radeloff et al. 2019); as these variables can predict the availability of resources for prey (Letnic and Ripple 2017) but also vegetative cover for hunting (Smith et al. 2020). To facilitate the interpretability of results between predictor variables, we scaled all variables 0 to 1 in a min–max transformation, and discarded those with high collinearity (Pearson’s *R* > 0.5), namely slope, minimum GPP and GPP variability. We extracted the landscape covariates value at the end of each step and created a conditional logistic regression model for each individual using the two-stage approach (Fieberg et al. 2010) to model the puma selection accounting for individual and site effects. Briefly, we compared a set of a priori candidate models—constructed by combining the aforementioned variables identified as important in univariate analyses into biologically plausible multivariate models (Table 1)—using the Akaike information criterion (AIC) for each individual and then selects the model with the lowest AIC average among the population. Then, for the most competitive model, we obtained the selection coefficients for each landscape variable and each individual in the five sites for day and night and their variance–covariance matrix. We used parametric bootstrapping (Fieberg et al. 2020) to resample each coefficient from a multivariate normal distribution with the original fitted coefficient and their variance–covariance matrix to obtain individual estimates for the population-level coefficient as the mean of each resampled coefficient across 2000 bootstrap iterations.

**Table 1:** Structure of the integrative step selection models tested using a conditional logistic approach for puma (*Puma concolor*) movement in five sites across the Americas

Model	AIC	ΔAIC	AIC_weight_
Elevation + Cumulative Gross Primary Production (GPP) + Distance to Water + Roughness + Human Footprint Index (HFI) + HFI:Time-of-day	7327.0	0.0	1.0
Elevation + Cumulative GPP + Distance to Water + Roughness + HFI	7343.1	16.1	0.0
Elevation + Cumulative GPP + Distance to Water + Slope + HFI	7344.2	17.3	0.0
Elevation + Cumulative GPP + Distance to Water + Roughness	7348.6	21.6	0.0
Elevation + Cumulative GPP + Distance to Water + HFI + HFI:Time-of-day	7352.0	25.0	0.0
Elevation + Cumulative GPP + Distance to Water + HFI	7368.5	41.5	0.0
Elevation + GPP Variability + Distance to Water	7374.2	47.2	0.0

All models included the movement variables: step length + ln(step length) + cos(turning angle). Time-of-day refers to day or night calculated for each specific end of the step. The response variable (Case) indicates whether a step was observed or available. For each observed step, ten available steps were randomly generated. AIC values show the average from all the individual puma models

### Diet

We used bulk ^13^C and ^15^N isotopes analysis to determine the diet and the niche width of pumas. Hair and feather samples were washed 3 times in a 2:1 mixture of chloroform:methanol and allowed to air dry at 40 °C (Pauli et al. 2009). Approximately 1 mg of tissue was homogenized, massed into tin capsules and analyzed with a Costech 4010 Elemental Combustion System attached to a Thermo Finnigan DeltaPLUS XP Continuous Flow Isotope Ratio Mass Spectrometer. Results are provided as per mil (‰) ratios relative to the international standards of Pee Dee Belemnite (VPDB; δ^13^C) and atmospheric nitrogen (AIR; δ^15^N) with calibrated internal laboratory standards. To compare the isotopic niche breadth between populations and their respective prey sources, we used ^13^C and ^15^N values to calculate the area (‰^2^) of Bayesian standard ellipses (SEA_B_) and standard ellipses corrected by small sample size (SEA_C_) using the *SIBER* package in R software (Jackson et al. 2011).

To estimate the relative contribution of prey to the diet of pumas, we used *MixSiar* package (Stock et al. 2018) for a Bayesian stable isotopes mixing model with Markov chain Monte Carlo (MCMC; chain length = 300,000; burn = 200,000; thin = 100; chains = 3), including the isotopic discrimination factor (δ^13^C = + 2.6‰; δ ^15^N = + 3.4‰, obtained for other carnivore, *Vulpes vulpes,* but proven effective on puma and other carnivore analysis (Roth and Hobson 2000; Magioli et al. 2014; Moss et al. 2016a)). We ran the model independently for each site using individuals as a fixed effect to estimate the individual diet and as a random effect to estimate the population diet. We used diet estimates obtained from kill sites and scats (Rodríguez Curras et al. 2022) as informative priors to the model and ran informed and uninformed priors. We categorized prey as domestic animals, large native animals (> 20 kg, Carbone et al. 2007) or small wild animals (< 20 kg, including native, synanthropic and invasive species). We combined prey that were in the same category but that were isotopically different a posteriori (Phillips et al. 2014; Stock et al. 2018). Finally, we calculated similarity and specialization indices (ranging from 0–1, where 1 represents high similarity and specialization, respectively) using the values of the Bayesian diet estimates for each individual (Newsome et al. 2012).

### Effects of landscape and movement on diet

To quantify the influence of landscape variables on the diet, we again used the HFI and the cumulative GPP. The cumulative GPP represents the total energy fixed by plants annually in a given area and can serve as an indicator of prey abundance (Letnic and Ripple 2017). We separated the HFI into agricultural footprint (i.e., cropland and pasturelands) and urban footprint (human population density, roads, railways, night-time lights and built environments). We used the home range of the GPS-collared individuals to extract the mean value for each landscape variable. As we could not obtain annual GPS data for pumas on two sites (Laguna Blanca and Uncompahgre Plateau), we used an 8 km radius as a circular buffer around the sample collection point, based on the average area of the other collared individuals.

We examined whether cumulative GPP, total HFI, urban HFI and agricultural HFI, home range size, nocturnality, HFI selection coefficients (obtained from previous step selection analysis), sex, age and subcontinent (North or South America) influenced diet specialization. We normalized all predictor variables with a min–max transformation to a 0–1 range. We used linear beta regression with a logit link function for all models with package *glmmTMB* (Brooks et al. 2017) in R. We compared a set of a priori candidate models identifying important variables in univariate analyses and combining them into biologically plausible multivariate models (Table 4). We selected the best model to predict diet specialization based on the lower Akaike’s information criterion value, corrected for small sample sizes (AIC_C_) and using maximum likelihood in the selection process. All variables we used have a variance inflation factor < 2, discarding multicollinearity. To account for the effect of the site we tested our top-ranked model with the site as a random effect.

## Results

### Home ranges, movement and temporal activity

**Fig. 2:**
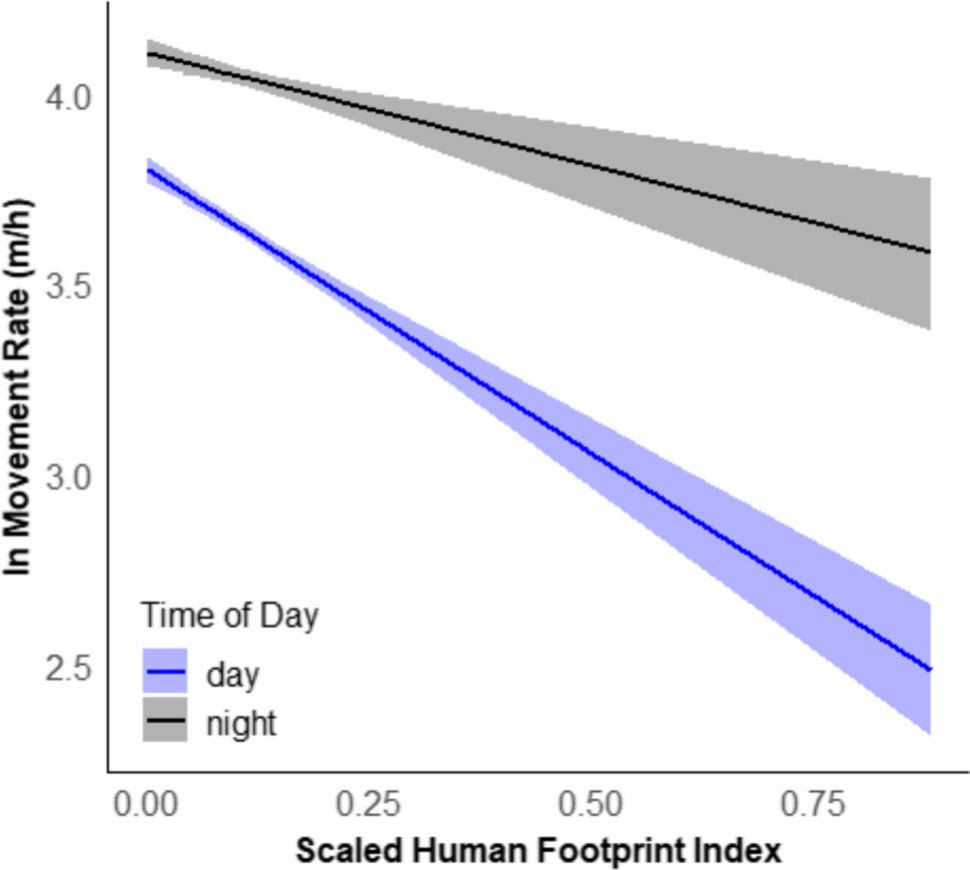
Predicted movement rate (ln) of pumas (*Puma concolor*) from five sites across the Americas in response to the HFI and the time of the day using a linear mixed model with site and individual ID as random factors ± 95% confident intervals

A total of 58 pumas were used in spatial analysis, which had > 6 months of monitoring with a total of 179,372 locations, leading to 104,523 steps within constant track sets. We found some evidence that the home range size of pumas decreased with HFI (*β* = −0.04, 95%CI [−0.09, 0.003]; *p* = 0.066; Figure S1). Likewise, within any site, the areas with higher HFI showed a reduction in the movement rate (*β* = −1.489 [−1.767, −1.205]; *p* < 0.001; Fig. 2). Pumas appeared to increase their turning angle to HFI (*β* = 0.84, [0.21, 1.51]; *p* = 0.023), but it did not change with the time of day (*β* = 0.02, [−0.08, 0.12]; *p* = 0.80), nor with the HFI and time interaction (β = − 0.23, [−1.06, 0.50]; *p* = 0.56). However, the time of day affected the movement rate. Specifically, the movement rate at night was higher (*β* = 0.3092 [0.265, 0.353]; *p* < 0.001) compared to daytime and additionally we found an interaction of time and HFI, indicating that the decline in movement rate in high HFI areas is less pronounced during the night (*β* = 0.8934, [0.545, 1.238]; *p* < 0.001; Fig. 2). We did not detect an association between nocturnality (i.e.: the proportion of distance traveled during the night) and the HFI (*β* = −0.01, [−0.04, 0.02]; *p* = 0.40). However, we did detect an effect of site, where the proportion of distance traveled at night in the most developed site (northern Front Range) was 45 [39, 51] %, compared to all other sites where the nocturnal movement was 60 [56, 64] % (*F*_4,53_ = 7.5, *p* < 0.001, Fig S2).

### Space use and selection

**Fig. 3:**
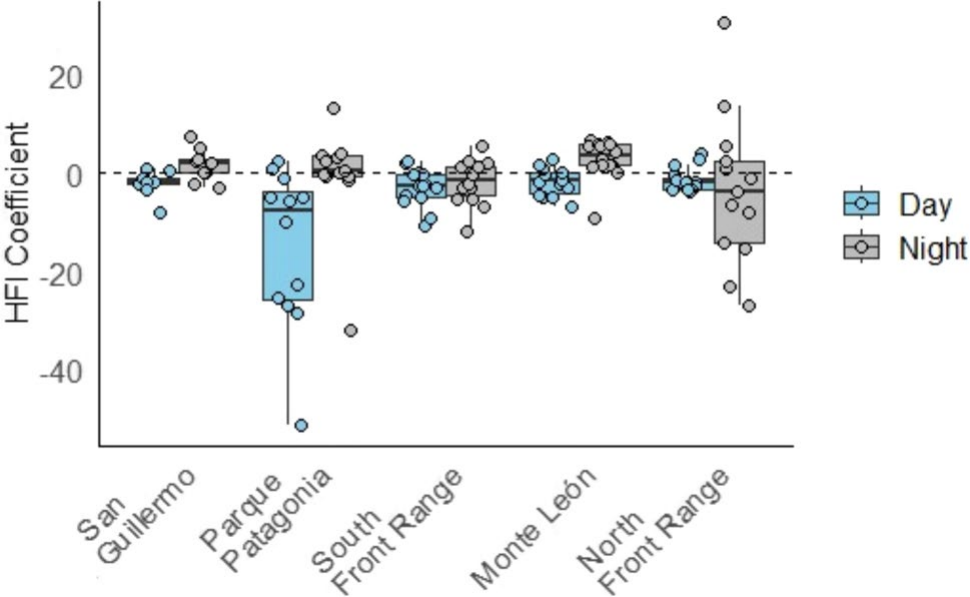
Selection coefficients of the integrative step selection analysis of the puma (*Puma concolor*) for the variable human footprint index (HFI) plotted from the day (grey) and night (light blue) models in the five different populations, where points represent the coefficient estimates of each individual and box plot the site population for the day and night

The iSSA model that best-explained movement steps included Elevation, Cumulative GPP, Distance to Water, Roughness, HFI, and the interaction of HFI and the time of day (Table 1). In decomposing the effects of specific components of the HFI index, we found that the total HFI was a better predictor to inform iSSA rather than any component by itself (Table S1). In general, pumas selected for roughness and distance to water, but against elevation and HFI (Table 2). However, these values varied when analyzed by site (Fig. 3). Nevertheless, pumas generally avoided areas with higher HFI, but that avoidance of HFI was attenuated during the night (Fig. 3).

**Table 2:** Selection coefficients (log-Resource Selection Strength) ± standard error showing habitat selection of pumas (*Puma concolor*) during day and night for different landscape features

Variable	Day	*p*	Night	*p*	Total	*p*
Elevation	**−0.24 ± 0.03**	< 0.001	0.05 ± 0.03	0.095	**−0.10 ± 0.02**	< 0.001
Cumulative GPP	**−0.37 ± 0.04**	< 0.001	0.08 ± 0.05	0.111	**−0.20 ± 0.03**	< 0.001
Distance to water	**0.18 ± 0.06**	0.003	0.00 ± 0.06	0.985	**0.12 ± 0.04**	0.003
Roughness	**0.21 ± 0.10**	0.036	**−0.52 ± 0.12**	< 0.001	**1.05 ± 0.07**	< 0.001
HFI	**−0.30 ± 0.04**	< 0.001	**0.69 ± 0.06**	< 0.001	−0.03 ± 0.04	0.453
HFI:time of day (night)	**-**		**-**		**0.40 ± 0.05**	< 0.001

Positive values represent preference, while negative values represent avoidance; in bold, significant values (p < 0.05)

*GPP* gross primary production, *HFI* human footprint index

### Diet

**Fig. 4:**
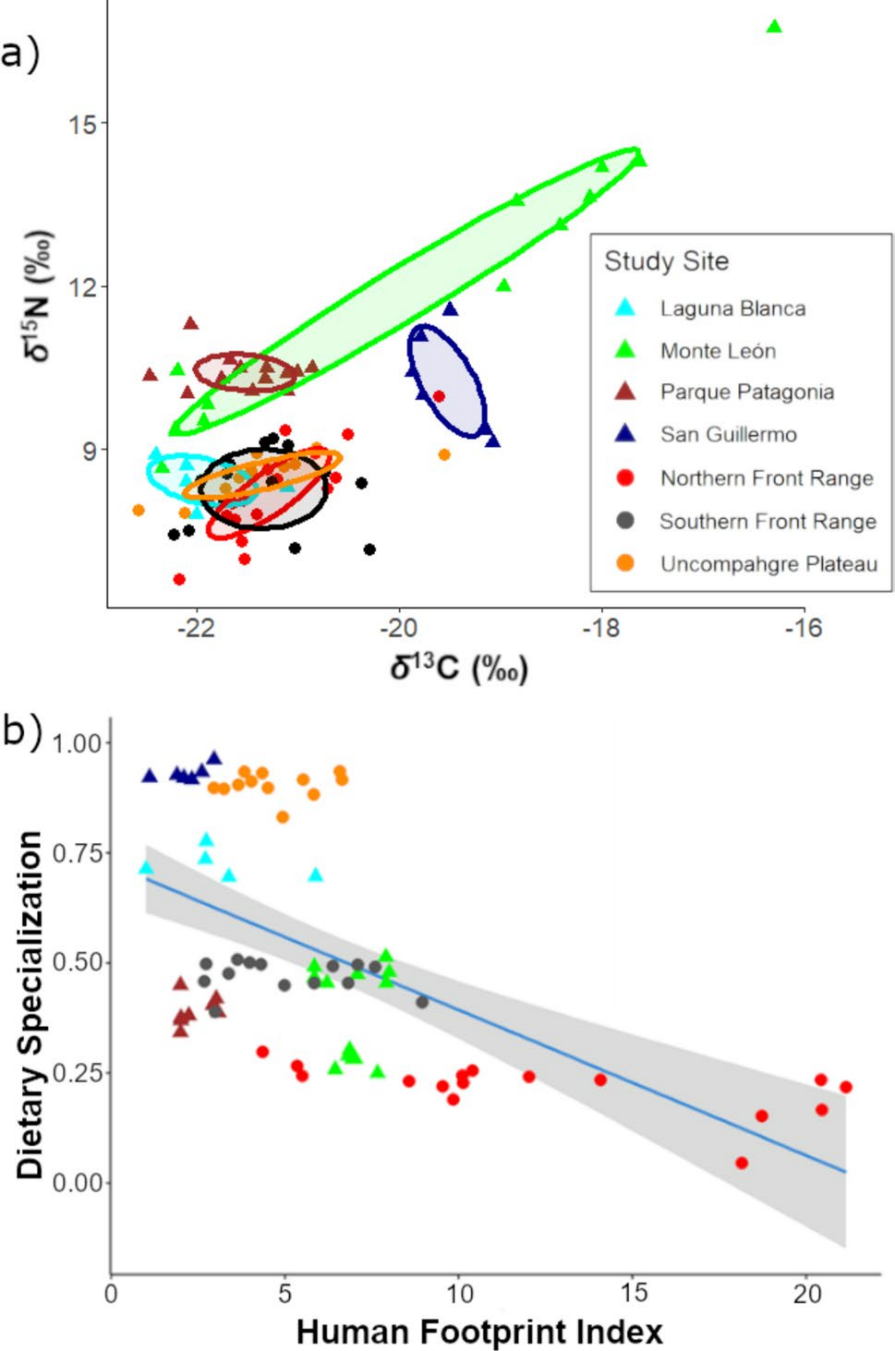
**a** Standard Ellipse Area corrected for sample size (SEAc) for each population of pumas (*Puma concolor)* on the seven different study sites using the ^13^C and ^15^N isotopic space. **b** Specialization index for each puma in response to the human footprint index (HFI; Venter et al. 2016) encompassing seven different populations in North (circles) and South America (triangles). The relationship between dietary specialization and the HFI is shown as a trend line (blue line) ± 95% CI (gray shaded area), *R*^*2*^ = 0.32, *p* < 0.001

Trophic niche width was similar among sites (SEA_B_ range = 0.64 to 1.45%^2^), except for the costal site of Monte León in South America, which exhibited a niche width at least > 4 times larger than the rest and driven principally by consumption of marine resources enriched in ^13^C (Fig. 4a). We also found a positive correlation between the predator ellipse area with the corresponding base-prey analyzed at the population level (*R*^*2*^ = 0.54, *p* = 0.034, *n* = 7, Table S2). Diet among individuals within each population showed high similarity, except for Monte León (*F*_6,76_ = 9.8, *p* < 0.001), where around half of the population preyed on Magellanic penguins (*Spheniscus magellanicus*). Pumas exhibited great variability in their diet depending on the site, consuming mainly native ungulates in 6 out of 7 sites (70 to > 95%), and in most cases supplementing the diet with domestic animals (0–20%), and small wild prey (0–40%, Table 3); the exceptional case occurred on Laguna Banca site, where native ungulates, the guanaco, have been completely replaced by livestock, primarily goats and sheep.

**Table 3:** Diet composition of pumas *(Puma concolor*) at the population level on seven different sites as the mean and 95% credible intervals

Study site	HFI	Informed prior			Uniform prior		
		Domestic prey	Large wild prey	Small wild prey	Domestic prey	Large wild prey	Small wild prey
		%	%	%	%	%	%
San Guillermo	2.2	0	91.9 [29–100]	8.1 [0–70]	0	41.4 [13–86]	58.6 [12–86]
Laguna Blanca	3.1	88.9 [36–100]	0	11.1 [0–64]	76.7 [25–99]	0	23.3 [1–75]
Parque Patagonia	2.4	12.7 [0–57]	81.6 [37–100]	5.7 [0–38]	36.9 [12–66]	43.1 [16–71]	18.0 [2–42]
Monte León	7.0	10.3 [0–41]	50.4 [5–85]	40.3 [12–90]	28.3 [3–65]	21.0 [2–61]	44.6 [19–81]
North front range	12.4	20.0 [3–43]	71.2 [57–85]	6.8 [0–18]	22.9 [6–39]	70.2 [57–85]	6.1 [0–18]
South front range	5.1	8.6 [1–22]	89.0 [76–97]	2.3 [0–8]	21.9 [6–43]	70.3 [53–83]	6.3 [0–20]
Uncompahgre plateau	4.7	1.2 [0–7]	96.9 [88–99]	1.9 [0–9]	15.2 [3–36]	80 [60–94]	3.5 [0–15]

Prey is categorized as domestic, including livestock and pets; large wild animals, corresponding to native ungulates; and small wild prey, which include wild prey weighing less than 20 kg. Priors are informed using kill site investigation or scats occurrence percentages

### Effects of landscape and movement on diet

Puma diet specialization decreased with increasing HFI overall (pseudo-*R*^*2*^ = 0.19, *β* = −1.96, 95%CI [−3.02, −0.88], *z* = −3.6 *p* < 0.001; Fig. 4b), which was a better predictor than urban footprint (*β* = −1.33, [−2.39, −0.28], *p* = 0.014) or agricultural footprint (*β* = −0.70, [−1.18, −0.22], *p* = 0.004) alone (Table 4). This top model also indicated that in South America pumas are less specialized (pseudo-*R*^*2*^ = 0.55, *β* = −2.21, [−2.86, −1.55], *z* =−6.6, *p* < 0.0001), while increasing cumulative GPP also decreases specialization (pseudo-*R*^*2*^ = 0.57, *β* = −4.19, [−5.43, −2.95], *z* = −6.6, *p* < 0.0001). We did not detect a relation of other variables, such as home range size or sex, with dietary specialization. When we included site effect as a random effect dietary specialization still decreased with HFI but to a lesser degree (*β* = −0.77, [−1.31, −0.24]; *p* = 0.0043, Figure S3).

**Table 4:** Results of model selection to predict the diet specialization index of each individual pumas (*Puma concolor)* across seven sites in the Americas using behavioral or landscape variables associated with each individual

Model	K	AIC_C_	Δ AIC_C_	AIC_C_ weight
Human Footprint (HFI) + Continent + Gross Primary Productivity (GPP)	5	−64.2	0	1
HFI + GPP + Nocturnality + Movement Rate	6	−44.4	19.7	0
HFI + GPP + Nocturnality	5	−42.5	21.7	0
HFI + Continent + GPP + Sex + Home Range	9	−39.4	24.8	0
HFI + GPP	4	−28.6	35.5	0
HFI	3	−27.1	37.0	0
Urban footprint	3	−21.4	42.8	0
GPP	3	−17.3	46.8	0
Nocturnality	3	−12.8	51.4	0
Home range	3	−12.8	51.4	0
Movement rate	3	−8.7	55.4	0
Null	2	−2.2	62.0	0

Models used the mean value within the home range of each puma to associate with landscapes features

## Discussion

Our findings highlight the plasticity of pumas in response to human disturbance in the temporal, spatial and trophic dimensions of their ecological niche. With increasing human disturbance, the rate of movement, and diet specialization of pumas decreased. Pumas inhabiting disturbed sites moved on average 44% less and consumed up to 20% more non-native prey. Contrary to our prediction, pumas inhabiting human-disturbed sites did not become more nocturnal. However, when considering movement as a function of both space and time, pumas, regardless of site, exhibited reduced movement during the day, especially pronounced in areas with higher human disturbance. Similarly, pumas avoided areas with high disturbance during the day but were neutral or even selected for them at night. Overall, pumas responded similarly to human disturbance, regardless of the site and the type of disturbance (i.e., the component of HFI), in their trophic, spatial and temporal niche axes, with an important interaction between the spatial and temporal components of the niche.

Movement reduction with increasing human disturbance aligns with previous studies (e.g., Tucker et al. 2018; Nickel et al. 2021) that have shown that pumas modify their paths and reduce the movement rate to navigate smaller and more fragmented habitats. Interestingly, reduced home ranges can also be associated with a preference for prey that is highly concentrated in small patches (Herfindal et al. 2005). This case occurs in one of our sites, Monte León, in which some pumas predate over a penguin colony (Serota et al. 2023). We also detect an increase in the turning angle in sites with higher human disturbance. This finding aligns with other studies that have found that pumas exhibit more sinuous travel paths in human landscapes, which is associated with elevated perceived risk due to higher traffic or activity in areas with high human density (Nickel et al. 2021). Our results confirm that pumas alter how far they move, and the characteristics of their paths in response to human disturbance. In general, these adjustments in reduced space use by pumas indicate that they consistently minimize exposure to human presence in the landscapes across sites independent of the main type of disturbance.

Surprisingly, we did not find a relationship between nocturnality and the mean HFI of a puma home range. Nocturnality has been documented as a strategy to avoid human encounters (Gaynor et al. 2018; Suraci et al. 2019); nevertheless, community dynamics play an important role in whether temporal shifts occur in carnivores (Frey et al. 2020). If pumas can avoid humans spatially, the need to change the diel activity is less likely (Van Cleave et al. 2018). Interestingly, the time of the day had an important effect over other variables, like movement rate and step selection in an interaction with HFI. Movement rate was considerably higher at night compared to during the day in areas with high HFI. This suggests that nocturnality could be observed at a finer scale, where each puma travels longer distances during the night when traversing areas of higher disturbance within their home range (Barocas et al. 2018). Similarly, this fine-scale association was observed when pumas showed a stronger avoidance of human features on the landscape during the daytime and even selection of those at night.

Our finding that pumas avoided human disturbance more during the day than the night reinforces the idea of a temporally dynamic response to humans and that pumas can be avoiding human landscapes when the likelihood of encountering humans is high. However, pumas may adjust their movement patterns not only to avoid human disturbance directly but also to align with the activity of their prey, which themselves may shift in response to human activity (Crawford et al. 2022). Fear of humans can operate at different levels and have indirect consequences on the ecosystem (Suraci et al. 2019), whereby the temporally dynamic response of pumas in human-disturbed landscapes could also be affecting the predator–prey interactions by either creating temporal refugees for prey (Berger 2007) or strengthening predation (Gilbert et al. 2022). Interestingly, the degree of avoidance varied among sites, likely reflecting local conditions. The attenuated avoidance, and in some cases selection, for areas featuring higher HFI at night in South America may be the result of multiple factors, including HFI being predominantly associated with pasturelands, rather than urban areas, in our South American sites. The selection of pumas for pasturelands in South America could also be a result of these lands featuring high densities of wild prey, like the guanaco, because of many ranches being abandoned in the last three decades (Novaro and Walker 2021) and, hence, HFI misclassifying the current state of those lands. Thus, the different responses in space selection that we observed between sites in North America versus South America could ultimately be an effect of the type of human disturbance that is most prevalent. Specifically, it appears that agricultural areas in South America either allow predator use at least during the night or offer land that can be more quickly recovered by wildlife. In contrast, pumas in North America exhibited avoidance of human-disturbed areas regardless of the time of day. Moreover, sites in North America contained proportionally less agriculture than our sites in South America but exhibited higher intensity use, potentially contributing to the more pronounced avoidance responses we observed in Colorado relative to the pasture-dominated areas of Argentina.

As expected, pumas exhibited great variability in their diet across their range in both South and North America. Large ungulates were the main prey, but pumas incorporated a variety of prey types depending on location. These results reinforce the concept that pumas are, indeed, generalist and opportunistic predators (Iriarte et al. 1990; Karandikar et al. 2022). Notably, we found that the HFI composite, regardless of the form of disturbance (i.e., urban vs. agricultural) can predict the degree of dietary specialization, particularly between sites. Specifically, pumas in more human-disturbed landscapes tended to exhibit a more generalized diet and consumed relatively fewer large ungulates. Other studies investigating pumas and carnivores in human-disturbed landscapes have also found plasticity in the foraging of predators, and an expansion of the niche to include domestic and synanthropic prey (Moss et al. 2016a; Ducatez et al. 2018). Our work reveals that this trophic plasticity is also driven by human disturbance and is consistent across continents. However, our observed increase in the generalist foraging strategies of pumas was not associated with changes in puma diel activity or space-use patterns. We suggest the diet is also driven by prey availability at each site, as the site was a relevant variable in our diet models. Specifically, human disturbance can increase domestic and synanthropic species, which can diversify prey assemblages up to a certain point and contribute to a reduced specialization in the predator diet (Manlick and Pauli 2020; Gámez et al. 2022); notwithstanding that in more disturbed scenarios native prey and biodiversity could be completely obliterated. Thus, pumas appear to adjust both their movement and their diet in the face of human disturbance; interestingly, however, space use was not a strong predictor of puma diet and specialization.

Taken together, our results demonstrate a generalized response of pumas to human disturbance in space use, diel activity and diet specialization. Nevertheless, this work also highlights the importance of local conditions in evaluating the effect of returning carnivores. For example, pumas inhabiting Laguna Blanca, a site featuring low HFI, exhibited high specialization despite native prey being completely replaced by exotic livestock. Even though puma diet specialization was high in Laguna Blanca, the functional role of pumas has dramatically changed, in terms of the high consumption of domesticated animals to the point of creating conflict with local ranchers (Pagnutti 2019; Rodríguez Curras et al. 2022). Similarly, the categorization between wildlands and pasturelands can be misinterpreted, particularly in sites in Argentina where livestock activities have been subject to variation in the last decades (Oliva et al. 2020). For example, the transformation of former ranchlands to national parks in the Argentinian Patagonia, or the opposite case, where livestock is inhabiting a national park in Laguna Blanca, can create scenarios in which the use of global indexes is not up to date or correctly classifying the landscape.

While our study provides a broad assessment of puma responses to human disturbance, there are important limitations to consider. First, HFI is useful for large-scale comparisons, but may not fully capture fine-scale habitat changes or shifting land use that will affect puma behavior (Woolmer et al. 2008). Additionally, it remains to be determined whether sites in South America with HFI comparable to the northern Front Range would exhibit ecological patterns comparable, particularly given the likely lower availability of large prey near densely populated cities in southern South America (Vynne et al. 2022). Moreover, our iSSA models were based on the population averages and may overlook important individual variation in behavior and space use, even when individual estimations are done, as the most important landscape variables driving puma behavior may be different between sites or individuals (Newsome et al. 2015; Nickel et al. 2020). Finally, we were not able to test other factors, like prey distribution and density, both of which are important component that shape predator movement patterns (Bartoń and Hovestadt 2013).

Ultimately, the responses that we have observed among pumas, as well as those documented among other carnivores, to human disturbance likely reverberate throughout the vertebrate community (Suraci et al. 2019). For example, the top predator avoidance of particular human-disturbed zones has contributed to mesopredators expanding into new areas (Frey et al. 2020), and increased activity of small mammals (Suraci et al. 2019). Similarly, the increased reliance of large carnivores on small prey (Moss et al. 2016a; Kuijper et al. 2016) may also affect the strength of interactions with their preferred prey. These changes can disrupt local food webs and result in altered ecosystem functions, broadening the implications of puma responses beyond immediate predator–prey interactions (Wirsing et al. 2021). The niche dynamics of predators in human-disturbed ecosystems will be important in determining their functional role in these emerging ecosystems, and their plasticity will likely modulate the strength of their top-down forces in human-disturbed landscapes. Notably, generalist foraging strategies should reduce top-down forces and the role of top predators in regulating prey populations (Ritchie et al. 2012; Allen et al. 2017). Thus, while the reintroduction of carnivores to relatively undisturbed areas could restore predator–prey interactions and associated ecological mechanisms, their return to human-disturbed landscapes may fail to restore ecological mechanisms typical of pre-disturbed states. Whether and to what degree reduced movement and dietary specialization within human-disturbed ecosystems ultimately alter predator–prey dynamics, trophic interactions, or competition is an important next step for future studies.

## Supplementary Information

Below is the link to the electronic supplementary material.

Supplementary file1 (DOCX 311 KB)

## Data Availability

The datasets used and analyzed during the current study will be available at 10.6084/m9.figshare.27179142 upon acceptance of this paper.
