# Supplementary material for: Human disturbance alters the foraging and spatiotemporal activity of a large carnivore
Source: Oecologia. 2025 Jun 26;207(7):112. doi: 10.1007/s00442-025-05752-x (PMC12202680; doi:10.1007/s00442-025-05752-x)
Supplement: Supplementary file 1 — Supplementary file1 (DOCX 311 KB) [file 442_2025_5752_MOESM1_ESM.docx]

## *Supplementary Material*

**Human disturbance alters the foraging and spatiotemporal activity of a large carnivore** Gonzalo Barceló^*^, Emiliano Donadio, Mathew W. Alldredge & Jonathan N. Pauli

**Results**

Given the high HFI in the North Front Range site, we also performed a regression analysis excluding that site. We found that diet specialization was still negatively correlated to the HFI (R^2^ = 0.11, β = -0.152, 95%CI [-0.26, -0.04], Figure S4).

**Tables**

**Table S1***:* Model selection from the components of the Human Footprint Index (HFI) for puma (*Puma concolor*) integrative step selection analysis in five sites across the Americas using the structure defined in the previous model selection Case ~ Elevation + Cumulative GPP + Distance to Water + Roughness + Human Variable + Human Variable:Time-of-day + step length + ln (step length) + cos(turning angle). AIC values show the average from all the individual puma models.

| Human Variable in Model | AIC | ΔAIC | AIC_weight_ |
| --- | --- | --- | --- |
| Total HFI | 7327.0 | 0.0 | 0.9 |
| Road Proximity | 7330.6 | 3.6 | 0.1 |
| Human Density | 7347.6 | 20.6 | 0.0 |
| Building Distance | 7347.9 | 20.9 | 0.0 |
| Agriculture Intensity | 7348.7 | 21.7 | 0.0 |

**Table S2**: Isotopic standard ellipses area (SEAc) for each puma (*Puma concolor*) population and their corresponding prey base and the ratio of the prey isospace in relation .

| **Population (n size)** | **S**EAc Pop Area (‰^2^) | **S**EAc Prey Area (‰^2^) | Proportion |
| --- | --- | --- | --- |
| San Guillermo (6) | 1.005 | 11.47 | 8.8 |
| Laguna Blanca (5) | 0.798 | 3.91 | 20.4 |
| Parque Patagonia (13) | 0.521 | 3.05 | 17.1 |
| Monte León (13) | 4.184 | 28.32 | 14.8 |
| Northern Front Range (20) | 0.866 | 17.46 | 5.0 |
| Southern Front Range (14) | 1.44 | 16.83 | 8.6 |
| Uncompahgre Plateau (12) | 0.64 | 16.19 | 4.0 |

**Figures**

*
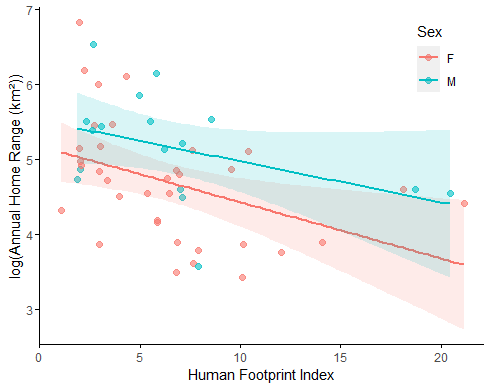
*

**
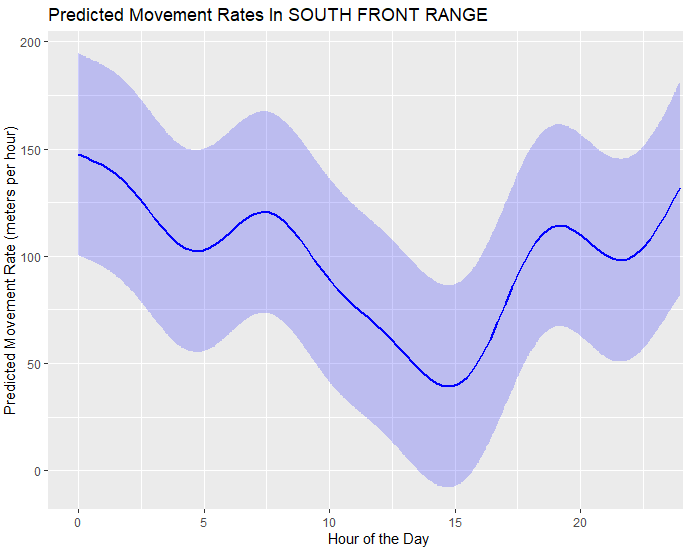
Figure S1**: Association between individual home range (HR) of pumas (*Puma concolor*) to the mean value of the human footprint index (HFI) within their respective home ranges. Trend lines represent the linear regression for males (blue) and females (females) and their respective 95% confidence interval.

a)

b)

| 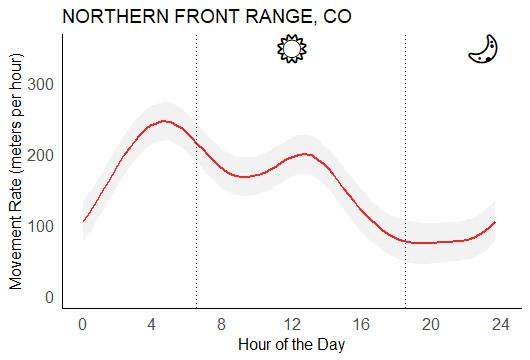 | 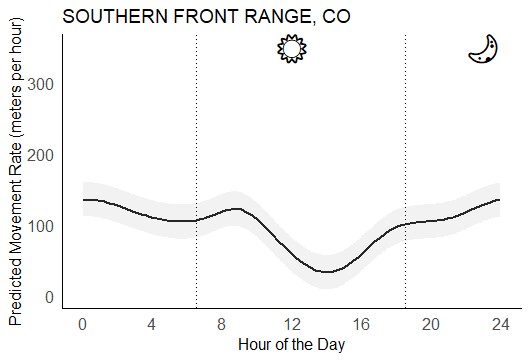 |
| --- | --- |
| 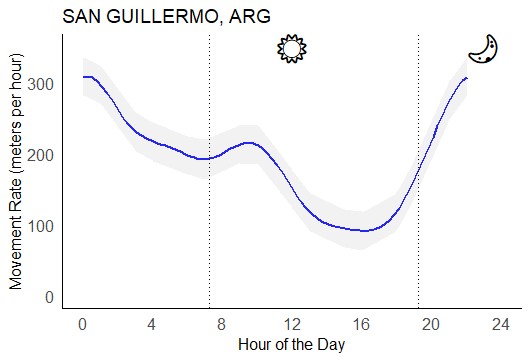  c) | 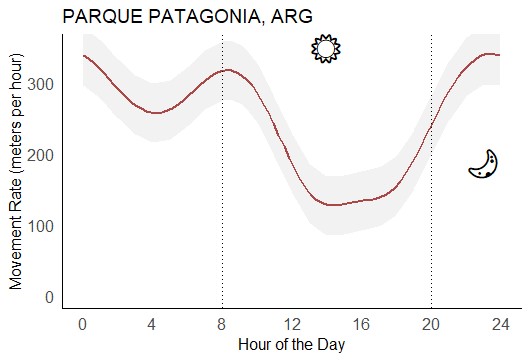  d) |
| 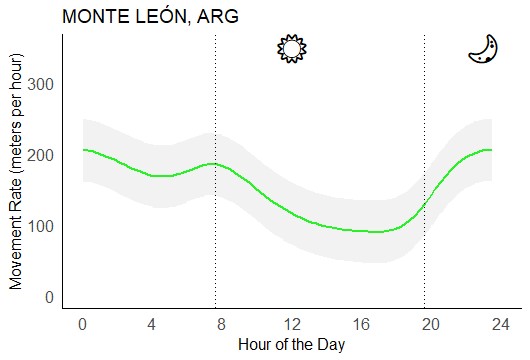  e) | 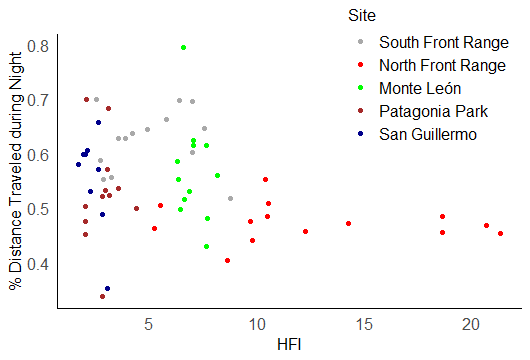  f) |

**
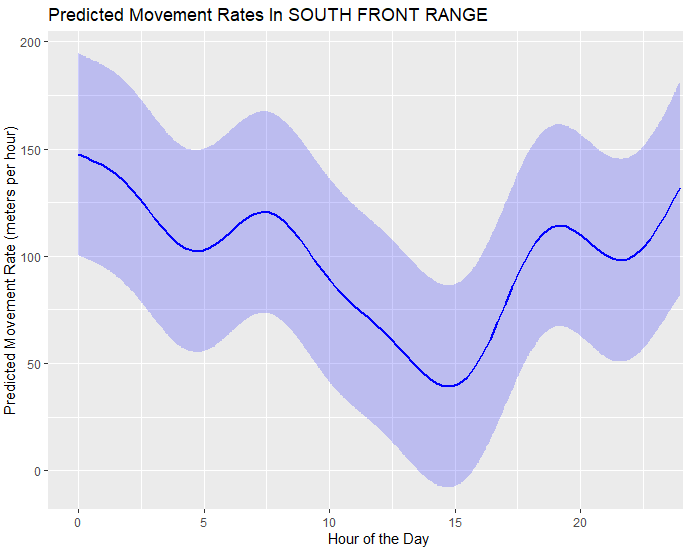
Figure S2**: a-e)Predicted daily movement rate (m/h) of the puma (*Puma concolor*) with 95% confidence intervals from five sites fitted with a generalized additive mixed model. Dotted lines represent the sunrise and sunset times at the equinox. f) Individual values of the proportional distance traveled during the night (nocturnality) and the mean human footprint index (HFI) value of the home range across the five sites.

a)

b)


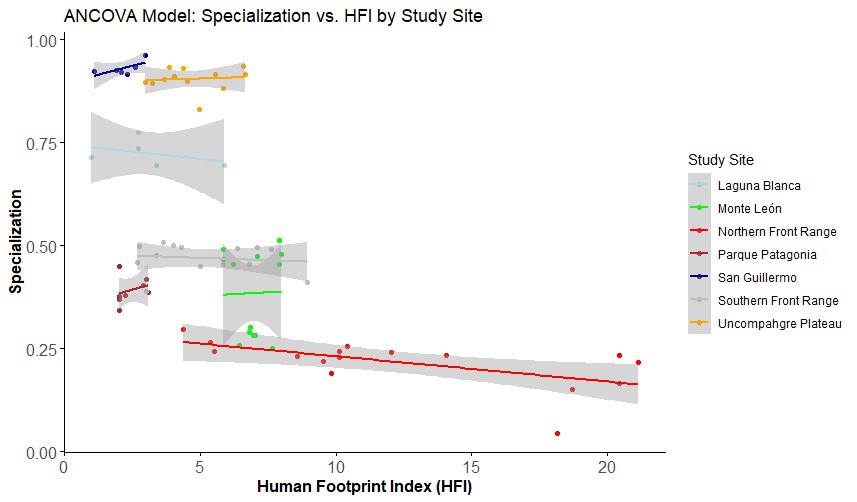


**Figure S3:** Diet specialization index for each puma in response to the human footprint index (Venter et al. 2006) encompassing seven different populations in North and South America . The relationship between dietary specialization and the human footprint index is shown as a trend line (solid line) ± 95% confident intervals (gray shaded area) per site.


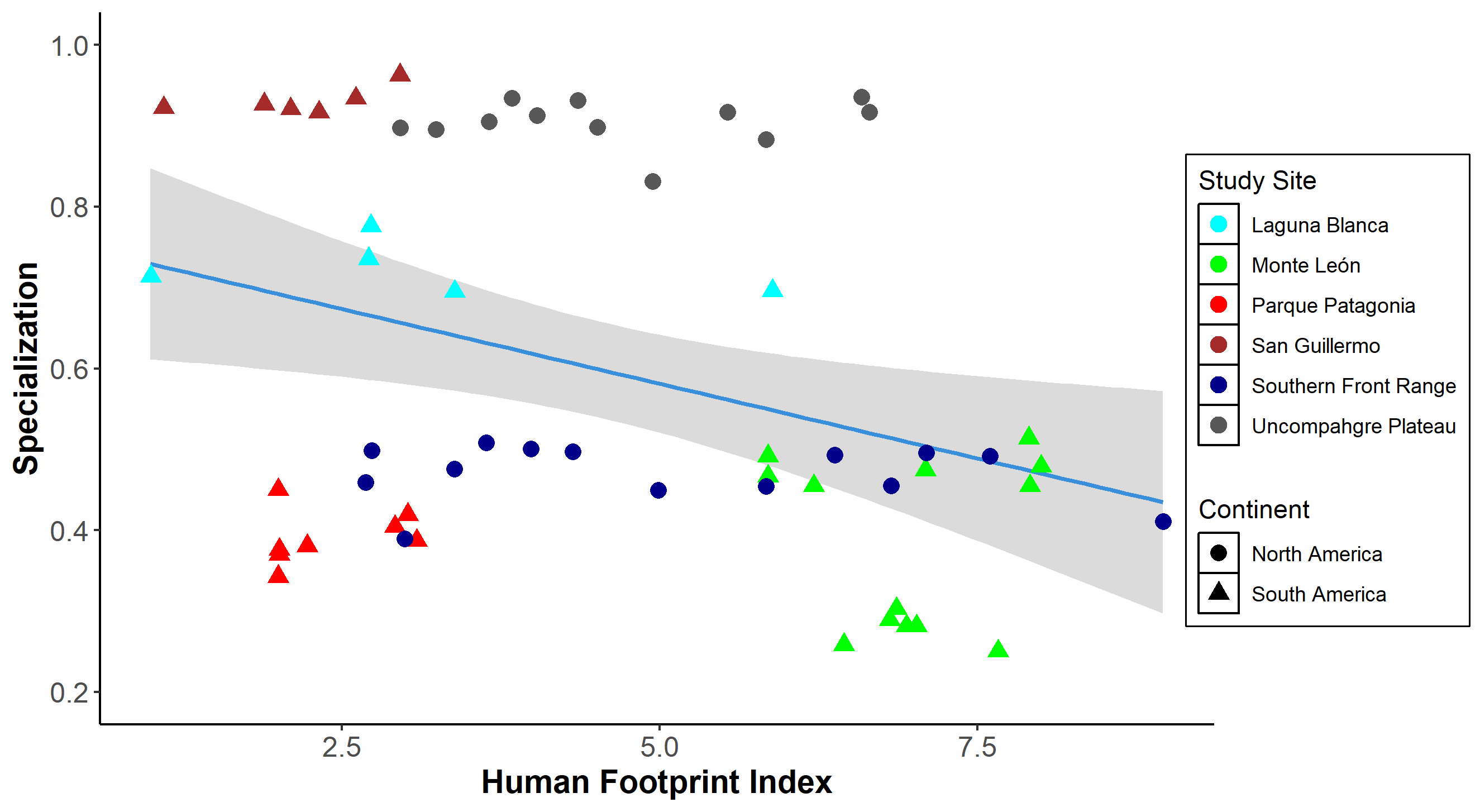


**Figure S4**: Diet specialization index for each puma in response to the human footprint index (Venter et al. 2006) encompassing six different populations in North (circles) and South America (triangles). The relationship between dietary specialization and the human footprint index is shown as a trend line (blue line) ± 95% confident intervals (gray shaded area), R^2^=0.11, p<0.01.
